# From promise to practice: Overcoming the barriers to unlock gerotherapeutics

**DOI:** 10.1016/j.jnha.2025.100639

**Published:** 2025-08-04

**Authors:** Emanuele Marzetti, Riccardo Calvani, Anna Picca

**Affiliations:** a Fondazione Policlinico Universitario “A. Gemelli” IRCCS, L.go A. Gemelli 8, 00168 Rome, Italy; b Department of Geriatrics, Orthopedics and Rheumatology, Università Cattolica del Sacro Cuore, L.go F. Vito 1, 00168 Rome, Italy; c Department of Medicine and Surgery, LUM University, Str. Statale 100 km 18, 70100, Casamassima, Italy

Aging is the primary risk factor for most chronic diseases, including cardiovascular disease, cancer, and neurodegeneration. Geroscience, a relatively novel discipline focused on understanding and targeting the biological processes of aging, seeks to extend healthspan rather than simply lifespan [1,2]. Yet, as highlighted in the scoping review by Muscedere et al. [3], no formal regulatory framework currently exists to guide the development or approval of gerotherapeutics. As a matter of fact, this hampers the translation of laboratory findings into clinical and public health benefits.

A central ethical and regulatory dilemma is whether aging should be recognized as a treatable condition or, more explicitly, as a disease [4]. Although aging is not classified as a disease by the World Health Organization (WHO) or other health authorities, its underlying biology is integral to the pathogenesis of several disease conditions, including cardiovascular disease, type 2 diabetes, Alzheimer’s disease, osteoarthritis, and many cancers [5]. This implies that intervening in aging pathways has the potential to delay or prevent multiple diseases simultaneously. Initiatives like the Targeting Aging with Metformin (TAME) trial epitomize efforts to circumvent existing regulatory gaps by focusing on composite endpoints, such as the delay of several chronic diseases [6]. However, without a formal regulatory classification of aging, therapies are forced into disease-specific pipelines that do not reflect their mechanisms of action or intended outcomes [[7], [8], [9]].

Several precedents from drug development in oncology and rare or emergent diseases offer potential models for accelerating gerotherapeutic development. In 2022, the US Food & Drug Administration (FDA) released an industry guidance document to provide recommendations on tissue agnostic drug development in oncology [10]. Accordingly, cancer therapies can be developed, evaluated, and approved for clinical use based on molecular targets rather than anatomical origin. Similarly, gerotherapeutics could be approved based on targeted biological mechanisms such as cellular senescence or mitochondrial dysfunction, irrespective of clinical manifestation. The FDA has also established an Accelerated Approval Program to allow expedited authorization of therapies intended to treat serious conditions and address unmet medical needs, based on surrogate endpoints (e.g., laboratory value, imaging finding, physiological measure) rather than conventional clinical outcome measures (e.g., disease remission) [11]. The implementation of such endpoints can substantially shorten the time required for regulatory clearance. The Sakigake program in Japan and the EU Adaptive Pathways similarly enable earlier access to high-need therapies. As highlighted in a recent expert consensus [12], aging fulfills many of the criteria applied in these frameworks: it represents an urgent unmet need, has modifiable mechanisms, and is suitable for mechanism-based targeting.

Muscedere et al. [3] also emphasize that progress in gerotherapeutics depends on the development and regulatory acceptance of biomarkers. These include measures of therapeutic mechanisms (e.g., senescent cell burden), biological age (e.g., DNA methylation clocks), and risk prediction (e.g., inflammatory markers). Functional measures such as gait speed and grip strength, which are predictive of health outcomes, should be considered valid surrogate endpoints [13,14]. Clinical trial methodology must also evolve. Most traditional trials are disease-specific and short-term, while aging is systemic and progresses over decades [15]. Innovative, “unconventional” endpoints, such as multimorbidity-free survival, resilience, or time to loss of intrinsic capacity, may be more appropriate [13,15,16,20].

In addition to scientific and regulatory challenges, economic constraints are a major barrier to the development of gerotherapeutics. These include long timelines, unclear reimbursement models, and limited patent exclusivity for repurposed drugs such as metformin or rapamycin [17]. Again, lessons from other therapeutic areas may help guide the path forward. For example, the Orphan Drug Act issued in 1983 created incentives for rare disease drug development through extended exclusivity, tax credits, and market protections [18]. Similar mechanisms could stimulate industry investment in gerotherapeutics. In addition, real-world evidence (RWE) platforms, such as the PROactive Solutions for Prolonging Resilience (PROSPR) program in the US [19], seek to collect data on resilience and functional aging to enhance understanding of biological drivers of age-related disease and facilitate the development of novel interventions to extend healthspan.

To be pragmatic, a stepwise, coordinated regulatory strategy is needed. In the short term, stakeholders must agree on core biomarkers and clinical outcome measures, and initiate adaptive, proof-of-concept trials using existing regulatory pathways (e.g., repurposing). In the medium term, pilot regulatory frameworks recognizing aging as a modifiable risk factor should be developed, incorporating RWE and health economics modeling. Finally, the long-term goal must be formal recognition of aging as a therapeutic indication through collaboration among international regulatory authorities.

To conclude, geroscience is at a crucial crossroad between promise and implementation. Scientific progress in understanding and targeting the biology of aging has advanced rapidly, while policy and regulatory frameworks have lagged behind, leaving promising interventions in a state of uncertainty. The scoping review by Muscedere et al. [3] offers a timely and comprehensive roadmap, outlining key barriers and possible solutions for gerotherapeutic development. While we acknowledge the ethical debate surrounding the “medicalization” of aging, gerotherapeutics explicitly aim to extend healthspan. The focus on function and quality of life can help mitigate concerns and align with the broader societal goal of healthier aging. In this scenario, the recognition of aging as a legitimate therapeutic target could instigate a paradigm shift in the prevention and management of chronic diseases, functional decline, and dependency in late life. The potential benefits, including healthier aging populations, reduced healthcare expenditures, and increased societal engagement among older adults, are too significant to ignore. The time has come for regulators, researchers, and policymakers to converge on the shared vision that aging, although inevitable, can and should be targeted through evidence-based interventions for the benefit of all.

## Declaration of Generative AI and AI-assisted technologies in the writing process

No Generative AI or AI -assisted technologies were used in the writing process.

## Funding

This work was partially funded by the Italian Ministry of Health (Ricerca Corrente 2025) and Next Generation EU in the context of the National Recovery and Resilience Plan, Investment PE8—Project Age-It: “Ageing Well in an Ageing Society”. This resource was co-financed by the Next Generation EU (DM 1557 11.10.2022). The views and opinions expressed are only those of the authors and do not necessarily reflect those of the European Union or the European Commission. Neither the European Union nor the European Commission can be held responsible for them.

## Declaration of competing interest

EM reports financial support was provided by Italian Ministry of Health. RC reports financial support was provided by European Commission. AP has no known competing financial interests or personal relationships that could have appeared to influence the work reported in this paper.
